# Supplementary material for: Unexplained repeated pregnancy loss is associated with altered perceptual and brain responses to men’s body-odor
Source: eLife. 2020 Sep 29;9:e55305. doi: 10.7554/eLife.55305 (PMC7524551; doi:10.7554/eLife.55305)
Supplement: Transparent reporting form [file elife-55305-transrepform.pdf]

1st Floor
24 Hills Road
Cambridge CB2 1JP, UK

P 01223 855340
W elifesciences.org
T @elife

eLife's transparent reporting form

We encourage authors to provide detailed information *within their submission* to facilitate the interpretation and replication of experiments. Authors can upload supporting documentation to indicate the use of appropriate reporting guidelines for health-related research (see EQUATOR Network), life science research (see the BioSharing Information Resource), or the ARRIVE guidelines for reporting work involving animal research. Where applicable, authors should refer to any relevant reporting standards documents in this form.

If you have any questions, please consult our Journal Policies and/or contact us: editorial@elifesciences.org.

Sample-size estimation

- You should state whether an appropriate sample size was computed when the study was being designed
- You should state the statistical method of sample size computation and any required assumptions
- If no explicit power analysis was used, you should describe how you decided what sample (replicate) size (number) to use

Please outline where this information can be found within the submission (e.g., sections or figure legends), or explain why this information doesn't apply to your submission:

Detailed information regarding sample size estimation can be found in the methods section. We conducted a power analysis for the main experiment (identification of spouse) which concluded a sample size of 29 subjects per group, therefore we recruited a pool of at least 30 participants per group for the entire set of studies. We matched the number of control women in each task to that of the RPL women. The number of women in each group, per experiment/task, is described in the main article, both in the results section (including main text and figure legends) and in the methods section.

Replicates

- You should report how often each experiment was performed
- You should include a definition of biological versus technical replication
- The data obtained should be provided and sufficient information should be provided to indicate the number of independent biological and/or technical replicates
- If you encountered any outliers, you should describe how these were handled
- Criteria for exclusion/inclusion of data should be clearly stated
- High-throughput sequence data should be uploaded before submission, with a private link for reviewers provided (these are available from both GEO and ArrayExpress)

Please outline where this information can be found within the submission (e.g., sections or figure legends), or explain why this information doesn't apply to your submission:

Information regarding performance of experiments and timing, repetitions, outliers, exclusion and inclusion of subjects and/or data can all be found in the methods section. Pre-study exclusions were as followed: for RPL group, women who don't experience a natural menstrual cycle. For control group, the same criteria as RPL was applied. In addition, women who experienced any type of pregnancy loss, including an abortion or ectopic pregnancy were excluded from the control group.

During data analyses, for each test, participants with Z-scores higher or lower than 2.7 STD from group's mean were excluded.

Statistical reporting

- Statistical analysis methods should be described and justified
- Raw data should be presented in figures whenever informative to do so (typically when N per group is less than 10)
- For each experiment, you should identify the statistical tests used, exact values of N, definitions of center, methods of multiple test correction, and dispersion and precision measures (e.g., mean, median, SD, SEM, confidence intervals; and, for the major substantive results, a measure of effect size (e.g., Pearson's r, Cohen's d)
- Report exact p-values wherever possible alongside the summary statistics and 95% confidence intervals. These should be reported for all key questions and not only when the p-value is less than 0.05.

Please outline where this information can be found within the submission (e.g., sections or figure legends), or explain why this information doesn't apply to your submission:

Statistical analysis methods, tests, assumptions and corrections, parameters, exact p-values, and effect sizes, can all be found both in the results section and methods section in the main paper. We show all individual participants in the graphs, even though well over 10.

(For large datasets, or papers with a very large number of statistical tests, you may upload a single table file with tests, Ns, etc., with reference to sections in the manuscript.)

Group allocation

- Indicate how samples were allocated into experimental groups (in the case of clinical studies, please specify allocation to treatment method); if randomization was used, please also state if restricted randomization was applied
- Indicate if masking was used during group allocation, data collection and/or data analysis

Please outline where this information can be found within the submission (e.g., sections or figure legends), or explain why this information doesn't apply to your submission:

Research samples in this study are comprised of two groups of women: women who experienced unexplained recurrent pregnancy losses (RPL), and women who never experienced a pregnancy loss (Controls), matched by age. All women in RPL group underwent clinical investigations independent of the present study, and were defined to have no known reason for their pregnancy losses.
Due to the nature of the study, no randomization was applied for subject selection. We did, however, randomize stimuli order within experiments, and counter-balanced conditions across subjects.
This information can be found in the methods section.

Additional data files ("source data")

- We encourage you to upload relevant additional data files, such as numerical data that are represented as a graph in a figure, or as a summary table
- Where provided, these should be in the most useful format, and they can be uploaded as "Source data" files linked to a main figure or table
- Include model definition files including the full list of parameters used
- Include code used for data analysis (e.g., R, MatLab)
- Avoid stating that data files are "available upon request"

Please indicate the figures or tables for which source data files have been provided:

eLife Sciences Publications, Ltd is a limited liability non-profit non-stock corporation incorporated in the State of Delaware, USA, with company number 5030732, and is registered in the UK with company number FC030576 and branch number BR015634 at the address 1st Floor, 24 Hills Road, Cambridge CB2 1JP | August 2014

All raw data for all figures but the MRI results is uploaded with this submission. All MRI data will all be posted on OpenNeuro upon publication.
